# Brain-reactive autoantibodies in neuropsychiatric systemic lupus erythematosus

**DOI:** 10.3389/fimmu.2023.1157149

**Published:** 2023-06-13

**Authors:** Cristina Cocco, Elias Manca, Giulia Corda, Maria Maddalena Angioni, Barbara Noli, Mattia Congia, Francesco Loy, Michela Isola, Elisabetta Chessa, Alberto Floris, Lorena Lorefice, Luca Saba, Alessandro Mathieu, Gian Luca Ferri, Alberto Cauli, Matteo Piga

**Affiliations:** ^1^ Department of Biomedical Sciences, University of Cagliari, Monserrato, Italy; ^2^ Rheumatology Unit, University Clinic, AOU Cagliari, Cagliari, Italy; ^3^ Department of Medical Sciences and Public health, University of Cagliari, Monserrato, Italy; ^4^ Multiple Sclerosis Center, Binaghi Hospital, ATS Sardegna, ASSL Cagliari, Cagliari, Italy; ^5^ Radiology Department, University Clinic, AOU Cagliari, Cagliari, Italy

**Keywords:** systemic lupus erythematosus, neuropsychiatric syndromes, autoantibodies, brain, neurons, immunofluorescence

## Abstract

**Introduction:**

The pathogenesis of neuropsychiatric systemic lupus erythematosus (NPSLE) is widely unknown, and the role of autoantibodies is still undetermined.

**Methods:**

To identify brain-reactive autoantibodies possibly related to NPSLE, immunofluorescence (IF) and transmission electron microscopy (TEM) on rat and human brains were performed. ELISA was used to reveal the presence of known circulating autoantibodies, while western blot (WB) was applied to characterize potential unknown autoantigen(s).

**Results:**

We enrolled 209 subjects, including patients affected by SLE (n=69), NPSLE (n=36), Multiple Sclerosis (MS, n=22), and 82 age- and gender-matched healthy donors (HD). Autoantibody reactivity by IF was observed in almost the entire rat brain (cortex, hippocampus, and cerebellum) using sera from NPSLE and SLE patients and was virtually negative in MS and HD. NPSLE showed higher prevalence (OR 2.4; p = 0.047), intensity, and titer of brain-reactive autoantibodies than SLE patients. Most of the patient sera with brain-reactive autoantibodies (75%) also stained human brains. Double staining experiments on rat brains mixing patients’ sera with antibodies directed against neuronal (NeuN) or glial markers showed autoantibody reactivity restricted to NeuN-containing neurons. Using TEM, the targets of brain-reactive autoantibodies were located in the nuclei and, to a lesser extent, in the cytoplasm and mitochondria. Given the high degree of colocalization between NeuN and brain-reactive autoantibodies, we assumed NeuN was a possible autoantigen. However, WB analysis with HEK293T cell lysates expressing or not expressing the gene encoding for NeuN protein (RIBFOX3) showed that patients’ sera carrying brain-reactive autoantibodies did not recognize the NeuN corresponding band size. Among the panel of NPSLE-associated autoantibodies (e.g., anti-NR2, anti-P-ribosomal protein, antiphospholipid) investigated by ELISA assay, only the anti-β2-glycoprotein-I (aβ2GPI) IgG was exclusively found in those sera containing brain-reactive autoantibodies.

**Conclusion:**

In conclusion, SLE and NPSLE patients possess brain-reactive autoantibodies but with higher frequency and titers found in NPSLE patients. Although many target antigens of brain-reactive autoantibodies are still undetermined, they likely include β2GPI.

## Introduction

Systemic Lupus Erythematosus (SLE) is a complex disease characterized by immune system dysregulation. The distinctive SLE characteristic is the production of self-reactive pathogenetic antibodies targeting any tissue or forming immune complexes (1). Moreover, SLE has a wide range of clinical manifestations, including neuropsychiatric (NP) symptoms affecting up to 90% of patients. NPSLE can be related to active disease (primary NPSLE) or organ damage and adverse drug effects (secondary NPSLE), but NP events can also be completely unrelated to SLE, representing accidentally co-occurring disorders (2). Several different manifestations can be observed among patients with NPSLE, and their severity may range from mild symptoms to more severe complications, even though NPSLE is generally associated with higher hospitalization rates and mortality (3, 4). The precise etiology of NPSLE has not been understood yet. However, NP events affecting the central nervous system (CNS) are usually differentiated between inflammatory and ischemic manifestations (5). Inflammatory mechanisms are related to known and unknown autoantibodies, proinflammatory cytokines, blood-brain barrier (BBB) dysfunction, and microangiopathy. In contrast, ischemic events are mainly related to antiphospholipid (aPL) autoantibodies and premature atherosclerosis (6). The attribution of neuropsychiatric events and the classification of the etiopathogenetic mechanisms underlying the individual manifestations of NPSLE is required for a correct therapeutic choice (7, 8). However, specific laboratory and neuroimaging biomarkers in NPSLE are scarce, which undermines the attribution process, especially for common events associated with quality of life impairment, such as seizures, mood disorders, cognitive dysfunction, and headache (9). Autoantibodies against neuronal auto-antigens, as well as other molecules, are among the most promising biomarkers. Circulating anti-P-ribosomal protein (anti-P) antibodies associated with lupus psychosis, depression, and executive dysfunction and their pathogenic mechanism in the brain likely involves perturbation of glutamatergic transmission and plasticity (10). Patients with NPSLE have shown antibodies to the DWEYS aminoacidic sequence (11). This sequence is found in the extracellular domain of murine and human N-methyl-D-aspartate receptor (NMDAR) subunits NR2a and NR2b (12). Animal model-based studies demonstrated that anti-NMDAR subunit 2 (anti-NR2) antibodies target the hippocampus of mice and lead to the impairment of both spatial cognition and spatial memory through aberrant excitatory signaling, apoptosis, dendritic pruning, and microglial activation (13, 14). The anti-NR2 antibodies may have the same effect in NPSLE patients with cognitive dysfunction. Besides cerebral ischemic events, seizures, cognitive impairment, psychosis, and depression were associated with aPL antibodies (15). However, none of the aforementioned studies can explain the whole spectrum of the neuropsychiatric manifestations observed among NPSLE patients (16).

This study aimed to investigate the presence of brain-reacting autoantibodies that could contribute to the picture of CNS manifestations in NPSLE patients. Our approach was based on immunohistochemistry (IHC) (through fluorescence and electron microscopy) to identify the brain areas, neuronal cell type/s, and subcellular areas targeted by the autoantibodies. Since humans and rats could only partially share common epitopes, we used brains from both species. In parallel, we used enzyme-linked immunosorbent assay (ELISA) to reveal the presence of known circulating autoantibodies correlating with the IHC data and western blot (WB) analysis to identify the possible molecular autoantibody targets.

## Materials and methods

### Human cohort

The human cohort enrolled in this study between January 2013 and February 2019 was composed of a total of 209 subjects, including 105 diagnosed with SLE (**Table 1**), 22 with multiple sclerosis (MS), and 82 (age- and gender-matched) healthy donors used as controls. The SLE cohort consisted of 36 patients with active inflammatory NPSLE, enrolled at the time of CNS events before starting the specific treatment, and 69 consecutive age and gender-matched SLE patients without active NP involvement enrolled in a 1:2 ratio. Diagnosis of SLE was performed according to the 1997 classification criteria proposed by the American College of Rheumatology (ACR) (17). Classification of NPSLE manifestation was based on the 1999 ACR nomenclature, and their attributions to SLE and inflammatory nature were defined by expert opinion. According to Zirkzee et al. (5), the classification of inflammatory NPSLE was supported by other organ active manifestations, C3 or C4 consumption, brain MRI abnormalities, response to immunosuppressive therapy, provided secondary NPSLE and ischemic NPSLE were excluded.

**Table 1:** Clinical characteristics and serologic abnormalities of the patients enrolled in the study.

	SLE (N = 69)	NPSLE (N =36)	p
**Sex ratio** (female: male)	63:6	33:3	
**Age, mean (yrs)**	43.2 ± 13.0	45.6 ± 14.4	0.377
**Disease duration, mean (yrs)**	8.6 ± 8.1	9.1 ±8.6	0.769
SLEDAI*	4.4 ± 5.4	13.9 ± 9.7	**<0.001**
**ANA ^1^ titer ≥ 1:80**	69 (100%)	36 (100%)	–
**ANA ^2^ titer ≥ 1:80**	63 (91.3%)	36 (100%)	–
**C3 (mg/dl)**	84.5 ± 26.1	65.2 ± 23.6	**<0.001**
**C4 (mg/dl)**	11.5 ± 8.6	8.3 ± 5.1	**0.043**
**Anti-dsDNA**	34 (49.3%)	23 (63.9%)	0.155
**Anti-Ro/SSa**	19 (27.5%)	16 (44.4%)	0.082
**Anti-La/SSB**	5 (7.2%)	5 (13.9%)	0.273
**Anti-Sm**	9 (13.0%)	11 (30.6%)	**0.031**
**Anti-RNP**	14 (20.3%)	12 (33.3%)	0.168
**Anti-P**	5 (7.2%)	11 (30.6%)	**0.002**
**Anti-DWEYS**	14 (20.3%)	15 (41.7%)	**0.020**
**Anti-cardiolipin IgG or IgM**	4/65 (6.2%)	5/35 (14.3%)	0.177
**Anti-Beta2-GPI IgG or IgM**	4/65 (6.2%)	5/34 (14.7%)	0.162
**Lupus Anticoagulant**	19/67 (28.4%)	8/35 (22.8%)	0.551
	**Inflammatory NPSLE manifestations**		
** Mood abnormalities**	–	10 (27.7%)	–
** Seizure**	–	6 (16.7%)	–
** Cognitive dysfunction**	–	6 (16.7%)	–
** Headache**	–	5 (13.9%)	–
** Psychosis**	–	4 (11.1%)	–
** Demyelinating syndrome**	–	3 (8.3%)	–
** Movement disorder (chorea)**	–	1 (2.8%)	–
** Acute confusional state**	–	1 (2.8%)	–

SLE, Systemic Lupus Erythematosus; NPSLE, Neuropsychiatric Systemic Lupus Erythematosus SLEDAI*, Systemic Lupus Erythematosus Disease Activity Index including NPSLE manifestations, SLEDAI values equal or higher than six is considered for active disease, ANA 1, anti-nuclear antibodies detected at the diagnosis using HEp-2 cells, ANA 2, anti-nuclear antibodies detected using HEp-2 cells at the sample collections, anti-dsDNA, anti-double strand DNA performed by Farr essay; Anti-P, anti-ribosomal-P protein; LA, lupus anticoagulant. Bold values are statistically significant.

Demographic data, clinical features, and ongoing treatments were recorded. SLE activity was assessed with the SLE Disease Activity Index 2000 (SLEDAI-2K). Organ damage was evaluated using the Systemic Lupus International Collaborating Clinics (SLICC) Damage Index (SDI). Blood samples were collected on 10 ml serum separator tube gel and centrifuged within one hour from the sample collection at 3,500 rpm for 15 minutes at room temperature. Then several aliquots were made and stored at -80°C.

A subgroup of patients underwent brain MRI 1.5 T examination within one month of sampling. Exams were performed on a 1.5 Tesla scanner (Philips Ingenia, Best, Netherlands) with a dedicated eight-channel neurovascular coil (transverse slices, 5 mm thick, axial orientation). Sequence parameters included isotropic DWI (with b value = 1000), multiplanar dual FSE proton attenuation, and T1WI, T2WI, FLAIR, and 3DT1W1. Brain MRI results were qualitatively classified as reported elsewhere (18).

### Rat and human tissues

Male Sprague Dawley rats (250–300g) were sacrificed by perfusion with 4% paraformaldehyde injected through the left ventricle. The rat brains were collected and stored in a 7% sucrose diluted phosphate buffer saline (PBS) at 4°C, included in optimal cutting temperature compound (19), then cut at 10 μm sections with the HM-560 cryo-microtome. The sections of the human post-mortem brain areas (provided by the New Castle Brain Resource, https://nbtr.ncl.ac.uk/), including the cerebellum (from 4 cases; 3 females and 1 male) and hippocampus (from 1 female case) used to perform the IHC were fixed, paraffin-embedded, and taken stored at -80 °C (autolysis time and pH were in a range of 16 – 81 hours and 6.47–6.96 respectively). Autoantibody reaction in the cerebellum was considered positive when immunostaining was revealed at least in 2 out of 3 human cases.

### Immunofluorescence

Rat sections were treated with 0.1% Triton-100 for 1 hour at room temperature and then incubated overnight at room temperature with human sera diluted (1:60 to 1:1,000) in a solution containing 3% normal rat, 3% normal donkey serum, and 3% phosphate buffer saline (PBS). The sera were detected using a Cy3-conjugated donkey anti-human antibody (1:500, Jackson ImmunoResearch). Sera were considered positive only when positivity was confirmed using at least three different rats at 1:60 dilution. The optical density (OD) of the fluorescent signal generated by each serum was measured with ImageJ. The OD of the healthy donors was used as the threshold to classify each SLE, NPSLE, or MS patient as positive or negative. Titers of the positive sera were performed by further dilutions up to 1:500,000. Double staining on rat sections was carried out by mixing the human sera with the commercial antibodies, such as rabbit anti-NeuN antibody (1:200; Abcam), mouse anti-VAChT (1:300; NeuroMab), mouse anti-vGlut1 (1:300; NeuroMab), chicken anti-GAD-67 (1:500; Abcam), rabbit anti-GFAP (Glial Fibrillary Acid Protein; 1:200, DABCO), and rabbit anti-MBP (Myelin Basic Protein; 1:100, Dako Products). Autoantibodies were revealed by using a Cy3-conjugated donkey anti-human antibody (1:500, Jackson ImmunoResearch), while the primary antibodies were revealed through Alexa488-conjugated donkey anti-mouse antibody (1:300, Jackson ImmunoResearch), Alexa488-conjugated donkey anti-rabbit antibody (1:300, Jackson ImmunoResearch), or Alexa488-conjugated donkey anti-chicken antibody (1:300, Jackson ImmunoResearch). The Hoechst-33342 was used for nuclei detection at the 1:2,000 dilution. The colocalization between autoantibodies and anti -NeuN, -GFAP, -MBP, -GAD-67, -vGlut1, and -VAChT antibodies was analyzed with Fiji (ImageJ Software) using the plug-in JACoP. Human brain sections were treated with 0.1% Triton-100 for 1 hour at room temperature and incubated overnight at 4 ^0^C with patient sera (1:200 dilution) in a solution containing 0.1% bovine serum albumin (BSA) in PBS. Sections of the cerebellum from each human case were incubated with 24 patient’s sera, equally subdivided between SLE and NPSLE patient groups (each including6 from both positive and negative sera in rat sections) as well as 2 sera from healthy donors and 2 from MS. Detection of the sera was performed by using a Cy3-conjugated donkey anti-human antibody (1:500, Jackson ImmunoResearch). An indirect immunofluorescence assay using HEP-2 cells substrate was used to determine serum antinuclear IgG antinuclear bodies (ANA). Fluorescence patterns were reported according to the International Consensus on ANA Patterns (ICAP) (20).

### Transmission electron microscopy

Rat cerebellum samples were cut into pieces (1 mm^3^) and fixed with a mixture of 3% paraformaldehyde and 0.1% glutaraldehyde in 0.1 M cacodylate buffer (pH 7.2) for 2 hours at room temperature. They were then rinsed in cacodylate buffer with 3.5% sucrose, dehydrated, and embedded in Epon Resin (Glycide Ether 100; Merck, Darmstadt, Germany). Semi-thin sections (thickness: 5 μm) were stained with toluidine blue and examined by light microscopy to check the histological appearance. Ultra-thin sections (thickness: 80-85 nm) were collected on nickel grids and processed for the IHC analysis. Before deposition of the sections, grids were hydrated with PBS solution in a moist chamber for 5 minutes. Next, the grids were treated with 1% BSA and 5% normal goat serum (NGS) in PBS solution to block non-specific binding. They were then incubated with human sera diluted 1:15, 1:20, 1:40, and 1:50 in 1% BSA and 5% NGS for 24 hours at 4°C. After rinsing in PBS, the grids were incubated for 1h at room temperature with the goat anti-human secondary antibody conjugated to 25 nm gold particles (Aurion, Wageningen, Nederlands) and diluted 1:50 in 1% BSA-PBS (21, 22). Some grids were also incubated with the anti-NeuN (MAB377, Merk) mouse primary antibody, diluted 1:25 and 1:50 in 1% BSA and 5% NGS, for 24 hours at 4°C. After rinsing in PBS, the grids were incubated for 1h at room temperature with the goat anti-mouse secondary antibody (Aurion, Wageningen, Nederlands) conjugated to 15 nm gold particles, diluted 1:50 in 1% BSA-PBS. After washing with PBS, grids were stained with uranyl acetate for 10 minutes and lead citrate for 2 minutes. Finally, they were observed in a JEOL 100S transmission electron microscope (TEM) operating at 80 kV and in a Jeol JEM 1400 Plus transmission electron microscope operating at 80 kV. Controls were incubated by omitting the primary antibody.

### Western blot

Western blot (WB) was performed using either HEK293T and *RBFOX3-*HEK293T cells transient overexpression lysates (Origene, catalogue number LC421190), which do not or do express the *RBFOXP3* gene (encoding the NeuN protein), respectively. Lysates were transferred to polyvinylidene difluoride (PVDF) membranes for 1 hour and 50 minutes at 200 Volt. The membranes were blocked with 10% BSA for one hour at room temperature and then incubated at 4°C for one night with human serum, including IHC-positive (2 from NPSLE and 1 from SLE) and 2 -negative sera diluted in a range between 1:8,000 to 1:50,000, and the rabbit anti-NeuN antibodies (Abcam), 1:200diluted. The membranes were washed three times with tris-buffered saline + 0.01% tween-20 (TBST). After washing, membranes were incubated at room temperature for 1 hour with the opportune secondary antibody (either donkey anti-human conjugated biotin or donkey anti-rabbit conjugated with biotin, both diluted at 1:10,000). The membranes were washed three times with TBST and incubated at room temperature for 30 minutes with streptavidin conjugated with horseradish peroxidase (HRP), diluted at 1:10,000, and finally washed 3 times with TBST. The antigen-antibody reaction was revealed using ThermoScientific^TM^Pierce Enhanced chemiluminescence WB Substrate. ImageQuant^TM^ LAS 4000 was used to detect the chemiluminescence.

### SLE-related serologic abnormalities

The serum quantification of C3 and C4 complement fractions, anti-ribosomal-P protein(anti-P; IBL international, RE70141), anti-Ro/SSA, anti-La/SSB, anti-RNP, anti-Sm (ENA kit, Dasit, Italy), anti-cardiolipin (aCL) IgG and IgM (Anticariolipine Bouty, Italy), anti-β2glicoproteinI (aβ2GPI) IgG and IgM (EUROIMMUN, Germany), was performed following the guidelines of the commercial ELISA kits. Anti-β2GPI and aCL IgG and IgM positivity was considered for titers higher than GPL and MPL 40 U/ml, respectively. The quantification of anti-NR2 antibodies was performed by homemade ELISA using the DWEYSVWLSN peptide (ThermoFisher, UK), as described by Putterman & Diamond (11). We used 10 healthy controls as a background and two standard deviations from the mean of healthy control optical density (OD) for selecting anti-NR2 positive sera. The OD was monitored at 405 nm using a multilabel plate reader (Chameleon: Hidex, Turku, Finland). Silica clotting time (HaemosilTM SilicaClotting Time, Instrumentation Laboratory, Milan, Italy) and dilute Russell’s viper venom time (HemosilTM dRVVT LAC screen and confirm, Instrumentation Laboratory, Milan, Italy) were used to detect lupus anticoagulant (LA) in the blood.

### Statistical analysis

Continuous variables are presented as mean (SD) or median and IQR. Absolute and relative frequencies are reported for categorical variables. For each IHC experimental set, the normality of data distributions was preliminary checked using the Goodness-of-fit test. The resulting *p* values were > 0.05 in all cases; hence, sample variances were measured with the F-Test for equality of variance, while individual or pooled variances were used for the two-tailed Student’s t-test. Pearson’s coefficient (r_p_) was used to measure the colocalization between the labeling patterns generated by sera autoantibodies and the other commercial antibodies used for this study. The result is +1 for perfect colocalization, 0 for no colocalization, -1 for perfect anti-colocalization, and >0.5 for good colocalization. The Chi-squared test, the Fisher exact test, and the Mann–Whitney U-test were used to compare data between NPSLE and SLE patients and between positive and negative patients for brain-reactive autoantibodies detection. Statistical analyses were carried out using the StatistiXL software. Odd ratio (OR) and 95% confidence intervals (CIs) are reported where relevant; *p*-values < 0.05 were considered to represent statistical significance.

## Results

### Autoantibody reactivity on rat brain

**Figure 1:**
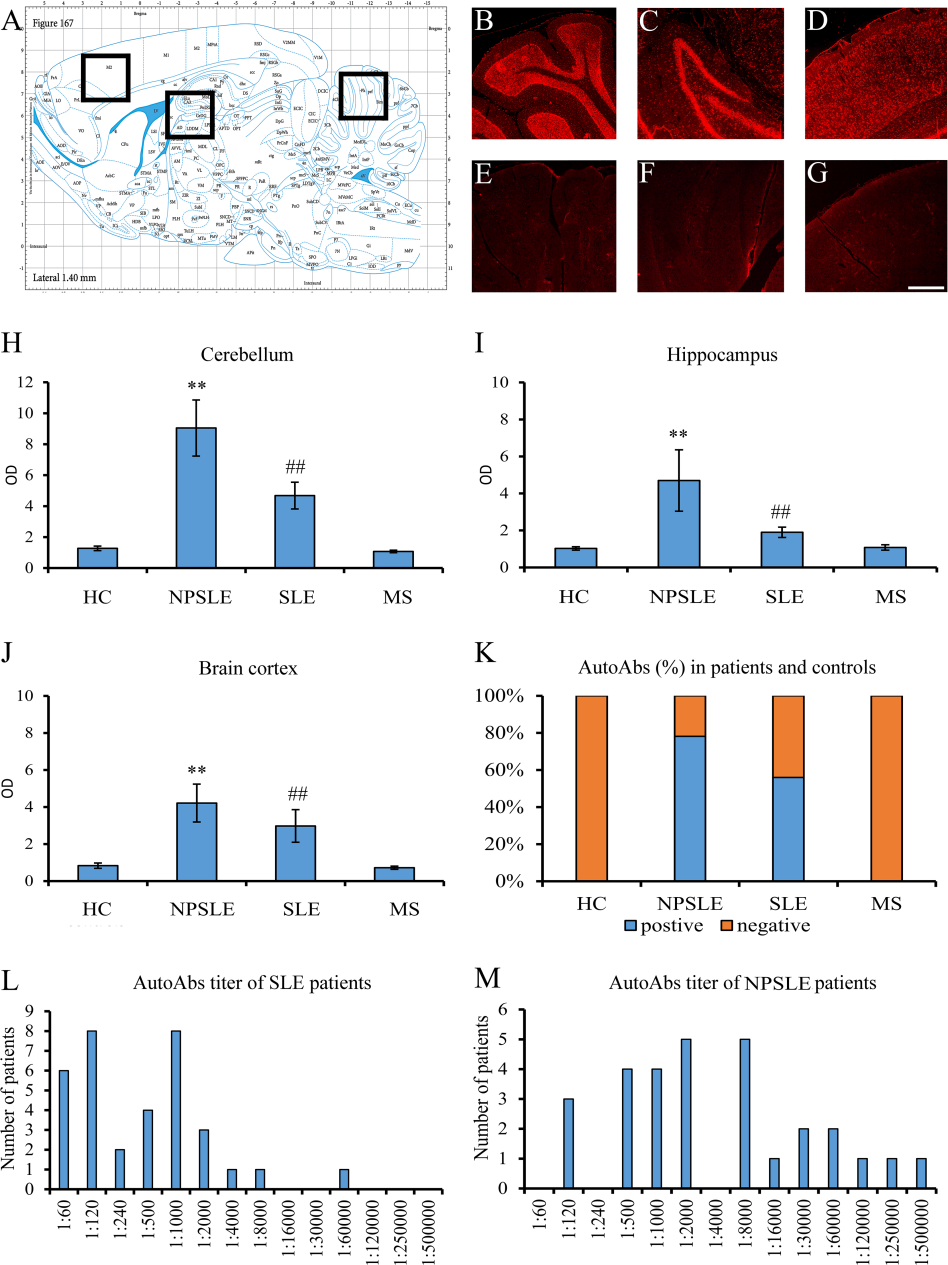
Brain reactive autoantibodies in NPSLE and SLE patients. The longitudinal rat brain is represented from the rat brain atlas (**A**: 6^th^ edition: by George Paxinos and Charls Watson Staining). Autoantibodies (identified with Cy3 red labeling) labeled the entire rat brain with brighter staining in the cerebellum **(B)**, hippocampus **(C)**, and brain cortex **(D)**. Sections incubated using diluent only were completely negative **(E–G)**. OD of the fluorescent signal obtained with NPSLE sera was higher than those obtained with HC, SLE, and MS sera in the cerebellum **(H)** and hippocampus **(I)** **(p<0.001: NPSLE vs. HC, MS, and SLE); ^##^(p<0.001: SLE vs. HC, MS). In the brain cortex, statistically significant values were obtained only between NPSLE or SLE vs. HC/MS but not between NPSLE vs. SLE **(J)**. The percentage of the rat-IHC positive sera in NPSLE patients was higher than in MS and HC **(K)**. Higher titers were revealed in NPSLE patients **(L)** compared to SLE **(M)**. Optical density (OD), Controls (HC), neuropsychiatric systemic lupus erythematosus (NPSLE), systemic lupus erythematosus (SLE), and multiple sclerosis (MS); Scale bar: 1 mm.

**Figure 2:**
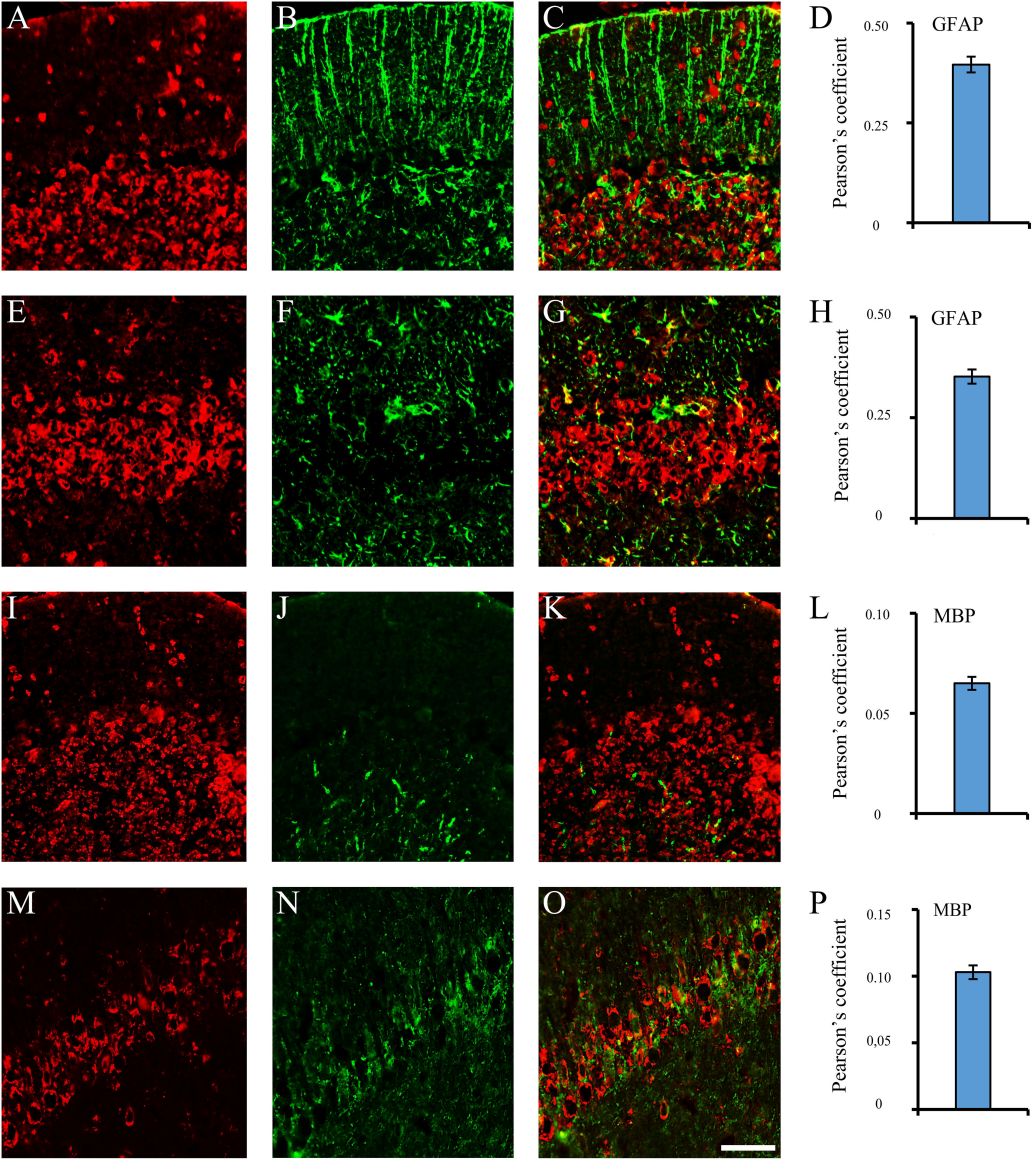
Brain reactive autoantibodies not decorating microglia. In the coronal sections of the cerebellum and hippocampus, staining patterns of the autoantibodies (**A, E, I, M**, red labeling) were compared with the anti: -GFAP (**B, J**, green labeling) and -MBP (**F, N**, green labeling) but colocalization was not observed (merge **C, G, K, O**: green + red labeling) as also shown by the Pearson’s coefficient (always <0.5). Cerebellum: **A–C, I–K**; hippocampus (CA1 cells): **E-G, M-O**. Autoantibody labeling was identified with Cy3 red labeling, while anti: -GFAP, -MBP antibodies were identified with Alexa488 green labeling. GFAP: glial fibrillary acidic protein; MBP: myelin basic protein. Scale bar: 0.1 mm.

**Figure 3:**
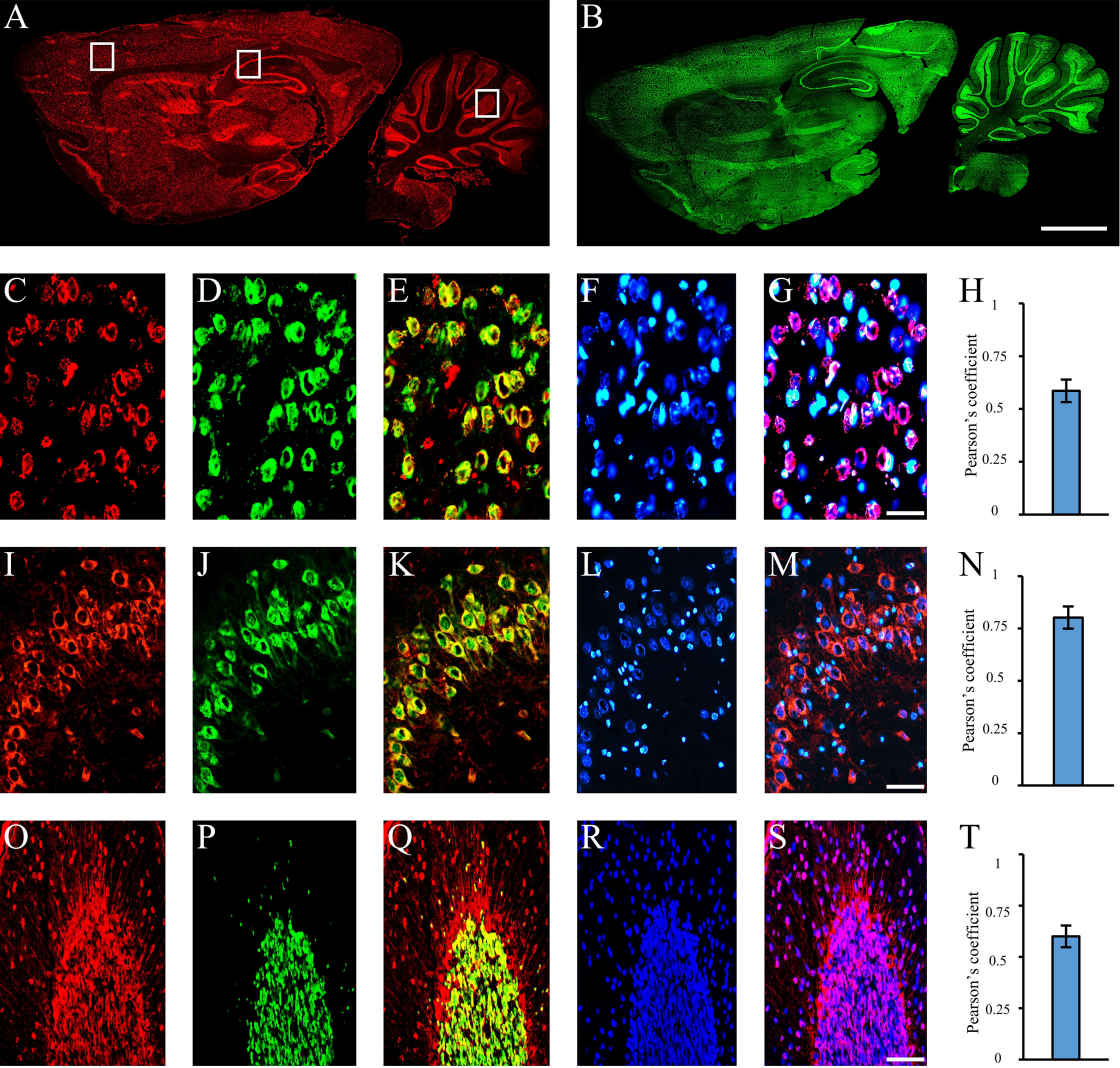
Autoantibodies staining NeuN-containing cells. Autoantibodies (**A**, red labeling) and anti-NeuN antibody (**B**, green labeling) on rat brain longitudinal section revealed a high degree of co-staining within the entire brain. Using coronal sections of the cortex **(C–G)**, hippocampus **(I–N)**, and cerebellum **(O–S)**, autoantibody labeling within brain cortex cells, hippocampal pyramidal and cerebellar granule cells (**C, I, O**: red labeling, respectively) was compared with the staining obtained with the anti-NeuN antibody (**D, J, P**, green labeling) and colocalization pattern was observed (**E, K, Q**: red + green labeling, colocalization pattern: yellow). Nucleus was identified by Hoechst-33342 dye (**F, L, R**: blue labeling) and used together with serum autoantibodies revealing autoantibody labeling within the nucleus (**G, S**, blue + red staining; colocalization pattern: violet) or cytoplasm (M, blue + red staining; colocalization pattern: violet). Pearson’s coefficient was always >0.5 **(H, N, T)**. Autoantibodies were identified with Cy3 red labeling, while anti-NeuN antibody with Alexa488 green labeling. Scale bars: 2 mm **(A, B)**; 0,1 mm for the others.

**Figure 4:**
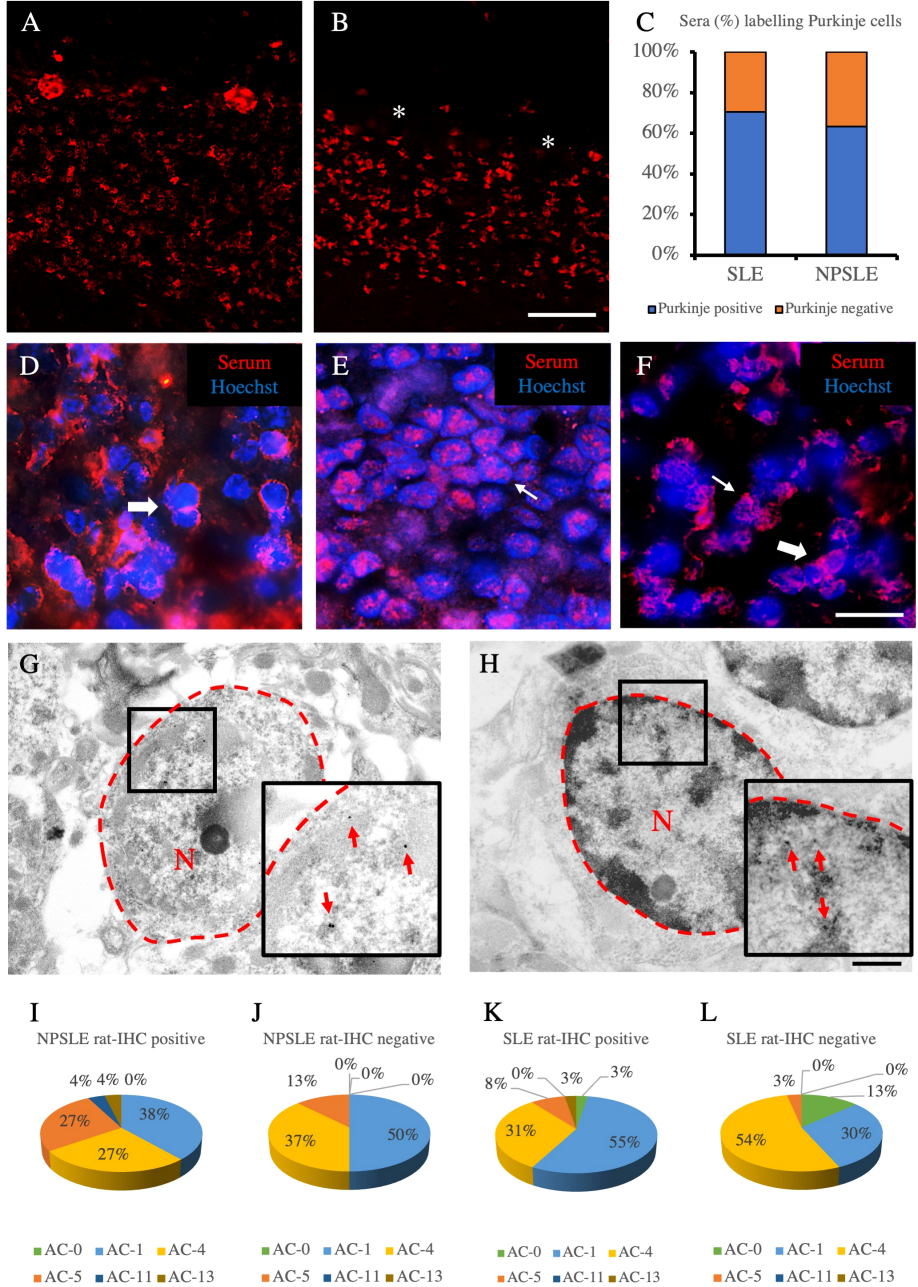
Autoantibody staining patterns within cerebellar and HEp-2 cells. Autoantibody staining patterns in rat cerebellum **(A–H)**. Purkinje cells were labeled **(A)** or not labeled (empty spaces identified by the asterisks) **(B)** by two different NPLSE patients’ sera. The percentage of sera labeling the Purkinje cells among SLE and NPSLE patients was equal to 70.4% and 63.2%, respectively **(C)**. Autoantibodies from NPSLE sera labeled cerebellar granular cells with a perinuclear **(D)**, or intranuclear **(E)** pattern, and sometimes both patterns were observed simultaneously **(F)**. Autoantibodies were identified with Cy3 red labeling. Nuclei were stained with Hoechst-33342 dye (blue). Scale bars: 0,2 mm **(A, B)**, 10μm **(C–F)**, 500nm **(G, H)**. Autoantibodies from a NPSLE patient’s serum **(G)** and the anti-NeuN antibody **(H)** labeled the nucleus (within the red dotted line) of the granule cells of the rat cerebellum, observed by the transmission electron microscope. The red arrows identify positive black spots. ANA patterns among the rat-IHC positive and negative sera **(I–L)**. The different HEp-2 staining patterns were obtained using the sera from NPSLE and SLE patients and were distributed almost equally among the rat-IHC positive and negative sera (patterns are classified according to the classification proposed by the international consensus on antinuclear antibody patterns) **(I–L)**. asterisk: Purkinjie cells.

Using sera from patients affected by SLE and NPSLE, autoantibody reactivity was detected in the entire rat brain. The most decorated areas were the cerebellum, hippocampus, and brain cortex (**Figures 1A–D**). No autoantibody reactivity was found using the diluent alone (**Figures 1E–G**) or the MS patient sera (**Figures 1H–K**). Between NPSLE and SLE groups, the higher optical density was found in NPSLE (**Figures 1H–J**), while between the brain areas, the cerebellum was the region with higher optical density (**Figure 1H**). The prevalence of sera showing rat brain-reactive autoantibodies was higher in NPSLE (27/36; 75.0%) than in SLE (38/69; 55.1%) patients (OR 2.4; 95%CI 1.1 to 5.9; p = 0.047) (**Figure 1K**). NPSLE patients also have higher titers (1:120 to 1:500,000 *versus* 1:60 and 1:60,000 in SLE serum patients; **Figures 1L, M**). In double experiments, while no co-staining pattern was observed using patient sera (from both SLE and NPSLE) and the anti-microglial marker antibodies (GFAP or -MBP: **Figures 2A–P**; both r_p_ < 0.5), NeuN antibody and brain-reactive autoantibodies stain the same cells (**Figures 3A–T**; r_p_ > 0.5), confirming that brain-reactive autoantibodies labeled neuronal targets. Colocalization profiles with NeuN were found within the entire brain cortex (virtually all layers), in the majority of pyramidal and granular cells of the hippocampus, and in almost all of the cerebellar granule cells (**Figure 3**). As to the specific staining patterns within the cells, autoantibody reactivity was found within the nucleus (and rarely within the cytoplasm) in the brain cortex (**Figures 3C–G**) and cerebellum (**Figures 3O–S**) or within the cytoplasm (and rarely in the nucleus) in the hippocampus (**Figures 3I–M**). Since the cerebellum was the region with higher OD values obtained using the NPSLE patient’ sera, we better focused on describing the autoantibody labeling in detail. In this area, autoantibodies brightly labeled the granule cells containing NeuN and weakly stained the Purkinje cells known not to contain NeuN (**Figures 4A–C**), but no other kinds of cells (**Supplementary Material 1**). Furthermore, within the granule cells, autoantibody reactivity was mainly found in the nucleus around the nuclear membrane (perinuclear staining) or within the nucleus with granular or mixed staining (**Figures 4D–F**). These patterns were simultaneously present, sometimes with a preponderance of one compared to the others. As the nucleus of these small cells was difficult to observe by fluorescence microscopy, we used TEM to reveal the subcellular staining of the autoantibodies. Using TEM, patients’ sera mainly labeled the nuclei of the granule cells within the heterochromatin and euchromatin and, to a lesser extent, the cytoplasm and mitochondria (**Figure 4G**). Reactivity was also detected in Purkinje cells, mainly within nuclei, in the RER, and to a lesser extent within cytoplasm and mitochondria (not shown). A similar staining pattern was observed using the anti-NeuN antibody in the granule cells (**Figure 4H**), while no labeling was observed in the Purkinje cells (not shown). Since the cerebellum revealed a heterogenous autoantibody staining pattern, we aimed to compare it with the ANA patterns obtained at the sampling time. Sera of NPLSE and SLE patients produced different HEp-2 staining patterns irrespectively of the presence of the rat-brain reactive autoantibodies (**Figures 4I–L**). The sera showing rat brain-reactive autoantibodies will be called rat-IHC positive (or negative).

### Autoantibody reactivity on human brain

**Figure 5:**
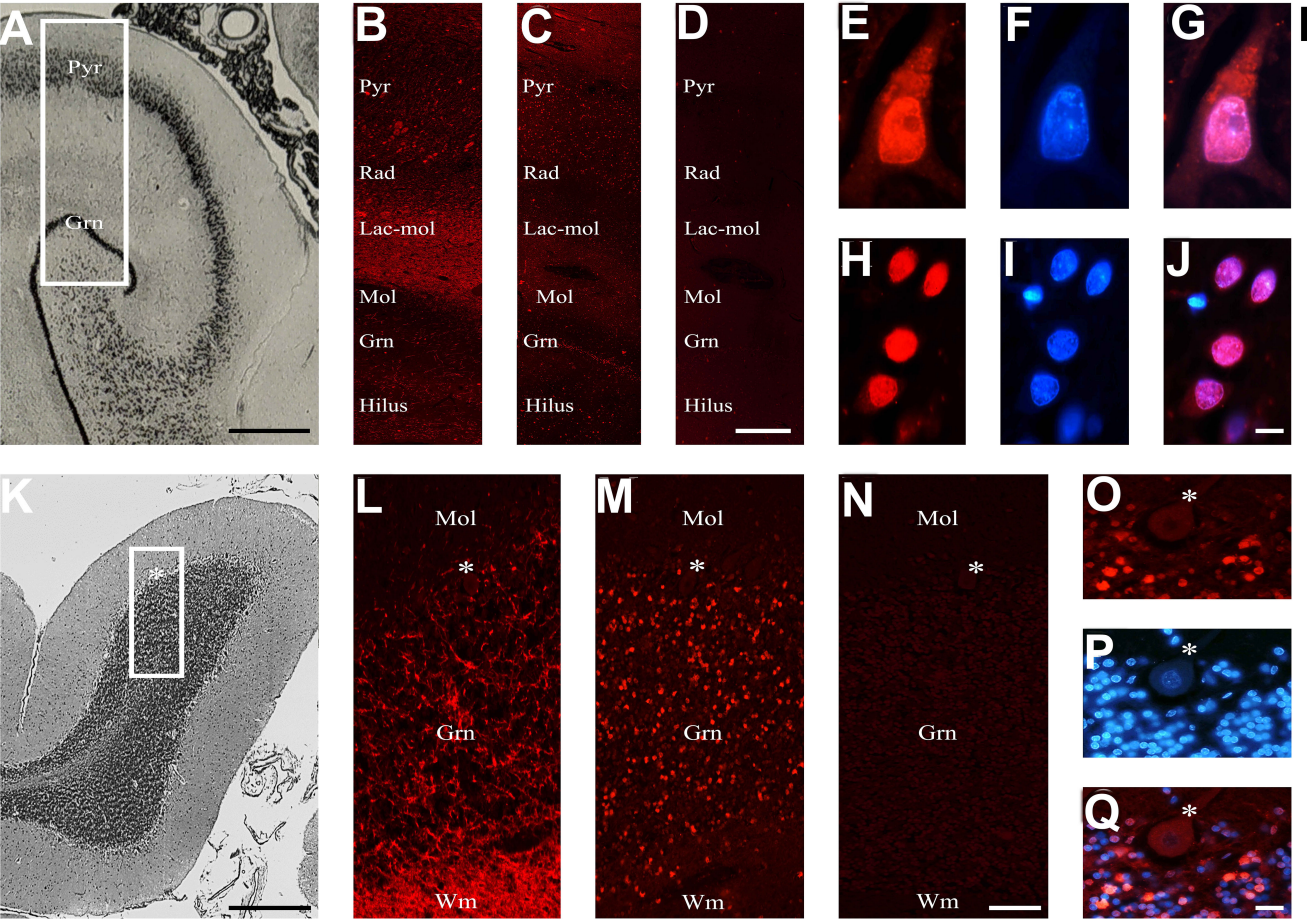
Brain reactive autoantibodies in the human brain. The human hippocampus is represented from the brain atlas **(A)**. The anti-chromogranin A antibody used as a positive control **(B)** had ubiquitous labeling, while the autoantibodies mainly labeled the Pyr and Gnr stratum **(C)**, and the section incubated using diluent was negative **(D)**; auto-antibody staining within the pyramidal **(E)** and granular cells **(H)** was very bright, and when nucleus was identified (**F, I**, respectively: blue labeling) with Hoechst-33342 **(F, I)** positive reactivity was observed within both the cytoplasm and the nucleus (red + blue staining: **G**; colocalization pattern: violet) or exclusively within the nucleus (red + blue staining: **J**; colocalization pattern: violet), respectively. The histology of the human cerebellar cortex is represented from the brain atlas. The anti-chromogranin A antibody used as a positive control brightly labeled wm and grn stratus **(L)**, while the autoantibodies mainly labeled the grn stratus **(M)**, and the section incubated using diluent was negative. Autoantibody reactivity on granule cells was very bright **(O)**. Nucleus was identified by Hoechst-33342 dye (**P**, blue staining). Autoantibody reactivity was found within the nucleus (red + blue staining: **Q**, colocalization pattern: violet). The human hippocampus and cerebellum are represented by the brain atlas (structure of the human brain, 3^thi^ edition, S.J DeArmond, et al.). Autoantibodies and anti-chromogranin A were identified with Cy3 red labeling, Hippocampus **(A-J)**: Pyr (stratum pyramidale), Rad (stratum radiatum), Lac-mol (stratum lacunosum-moleculare) Mol (stratum moleculare), Gnr (granular cells of the dentate gyrus). Cerebellum **(K–Q)**: mol: molecular layer, grn: granular cell layer, asterisk: Purkinje cells, wm: white matter. Scale bars: 5 mm **(A)**, 1 mm **(C)**, 10 μm **(I)**, 5 mm **(J)**, 1 mm **(L)**, 10 μm **(O)**, 10 μm **(X)**.

To corroborate the results obtained in rats, we studied, through the human cerebellum and hippocampus, selected human sera among those that proved positive or negative in rat brains (**Figures 5A–O**). The preservation of the human brain tissues was checked by using an antibody anti-chromogranin A, which was always positive and whose localization was compatible with its expression in the brain (**Figures 5B, L**). A positive reaction was observed in 75% and 67% of the rat-IHC positive sera from NPSLE and SLE patients, respectively. In both brain areas, a positive autoantibody reaction was observed only using the rat-IHC positive sera from both SLE and NPSLE patients (**Figures 5C, M**) but not using the rat-IHC negative sera (**Figures 5D, N**). Positive sera labeled both the cytoplasm and nucleus of hippocampus neurons (**Figures 5E–G; H–J**) as well as the nucleus of the cerebellar granule cells (**Figures 5O–Q**), whereas the Purkinje cells were never labeled by the rat-IHC positive sera (**Figure 5M**). Unfortunately, negative staining was revealed using the NeuN antibody, suggesting the susceptibility of this molecule to post-mortem autolysis (not shown).

### Searching for molecular targets of brain-reactive autoantibodies

**Figure 6:**
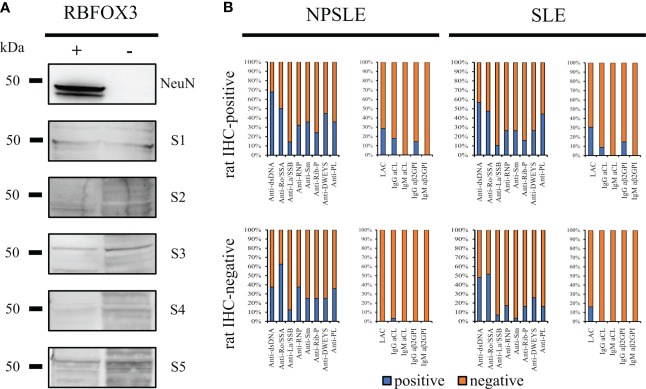
Searching for autoantibody targets. NeuN is not a brain-reactive autoantibodies target **(A)** HEK293T cells were transfected either with a vector containing the RBFOX3 gene (+) or with an empty vector (-). Anti-NeuN antibody recognized the corresponding bands (48/46 kDa) on transfected HEK293T cells only, while the autoantibodies from five different patients’ sera with SLE and NPSLE (3 IHC-positive and 2 IHC-negative sera) did not recognize a corresponding band. The bar charts show the percentage of the known autoantibodies in the rat-IHC positive and negative sera from NPSLE and SLE patients **(B)**. Anti -dsDNA, -Ro/SSA, -La/SSB, RNP, -Sm, -Rib-P, -DWEYS, were present in both rat IHC positive and negative sera instead the anti-PL including LAC, -IgG CL, -IgM Cl, -IgG ß2GP1, -IgM ß2GP1 were virtually negative in the rat-IHC negative sera from NPSLE.

Considering the wide co-staining pattern between the anti-NeuN antibody and the rat-IHC positive sera, we performed using HEK293T cell lysates which do not or do express the *RBFOXP3* gene (encoding the NeuN protein). None of the rat-IHC positive sera did recognize the bands corresponding to the NeuN protein using *RBFOX3-*HEK293T cell lysates (**Figure 6A**).

Assuming that the molecular target of the autoantibodies contained in the rat-IHC positive sera could be represented by autoantibodies known for their association with NPSLE and SLE (anti-dsDNA, -Ro/SSA, -La/SSB, -RNP, -Sm, -Rib-P, and -DWEYS) their distribution was investigated. All these autoantibodies were present in both the rat-IHC positive and negative sera, suggesting they were not responsible for the labeling we observed in rat brain (**Figure 6B**). However, we found that the aβ2GPI IgG were present in the rat-positive IHC sera and were absent in the negative ones, suggesting that they might be part of the heterogeneous autoantibody population responsible for the labeling we observed in rat brain(**Figure 6B**).

### Brain-reactive autoantibodies and SLE neuropsychiatric manifestations

**Figure 7:**
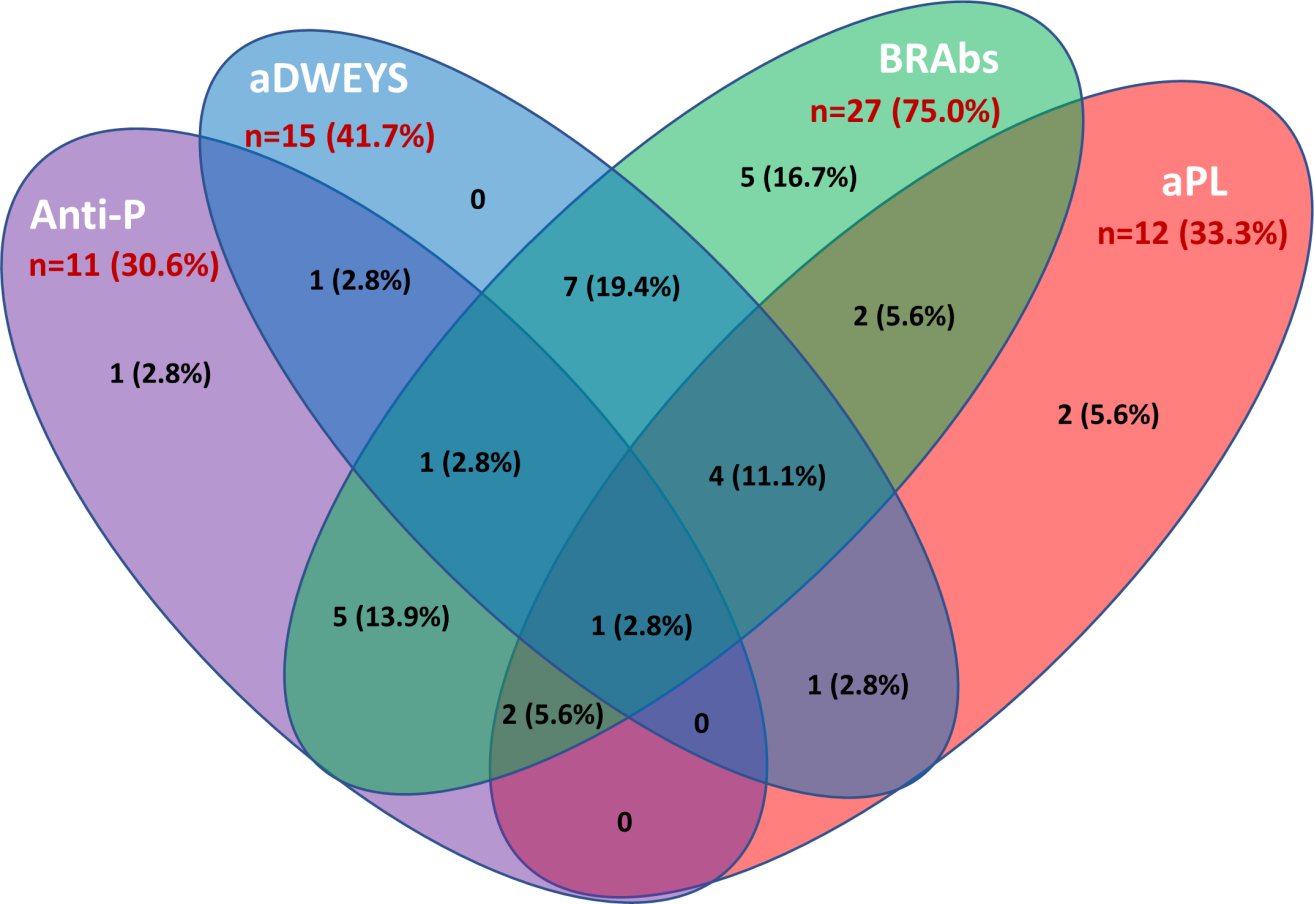
Autoantibodies associated with inflammatory NPSLE. The Venn diagram shows the number and percentage of positive autoantibodies from the group of NPSLE patients and highlights the possible overlap of the autoantibodies considered relevant in the inflammatory involvement observed in SLE patients. Among 36 patients with NPSLE, only 4 were seronegative for these autoantibodies. Anti-P: anti-P-ribosomal protein. aDWEYS: antibodies to the aminoacidic sequence DWEYS found in the murine and human N-methyl-D-aspartate (NMDA) receptor subunits NR2. BRAbs: brain-reactive autoantibodies. aPL: antiphospholipid antibodies.

The inflammatory NP manifestations developed by the NPSLE patients enrolled in the study and the associated serologic abnormalities are reported in **Table 1**. Besides a higher prevalence and titer of brain-reactive antibodies, NPSLE patients had lower C3 (p <0.001) and C4 (p = 0.043) complement fractions serum levels and higher incidence of anti-P (p = 0.002), anti-NR2 (p = 0.020) and anti-Sm (p = 0.031) antibodies than SLE patients. Only 4 out of 36 NPSLE patients were seronegative for autoantibodies known to be associated with CNS involvement (**Figure 7**). Brain-reactive autoantibodies were not associated with specific NPSLE manifestations, and the 9 patients who showed rat-IHC-negative sera suffered from cognitive dysfunction (n=2), seizure (n=2), psychosis (n=1), depression (n = 1), maniac disorder (n=1), headache (n=1), and MS-like syndrome (n=1). Median SLEDAI score was higher in patients positive (6; IQR 2 – 11.8) than in those negative (4; IQR 0.2 - 8) for brain-reactive autoantibodies (p = 0.029), whereas the distribution of age, disease duration, gender, and SDI did not differ between groups.

Brain MRI was performed in 69 patients (24 NPSLE and 45 SLE), but no significant difference in the distribution of MRI abnormalities according to brain-reactive autoantibodies status was detected between groups (**Table 2**). Grey matter hyperintensity (GMHI) lesions were more frequent in patients showing positive brain-reactive autoantibodies (11.9% vs. 0%; p = 0.064), whereas diffuse brain atrophy was more frequent in those negative (2.4% vs. 14.8%; p = 0.053), although not statistically significant. Separately analyzing brain MRI findings in NPSLE patients, GMHI were exclusively found in NPSLE patients with brain-reactive autoantibodies (5 out of 19, 26,3%), but their prevalence did not statistically differ from NPSLE patients negative for brain-reactive autoantibodies (0 out of 5, 0%; p = 0.554). On the other hand, 3 out of 5 (60%) NPSLE patients negative for brain-reactive autoantibodies showed diffuse brain atrophy compared to 0 out of 19 (0%) NPSLE patients carrying brain-reactive autoantibodies (p = 0.004).

**Table 2:** Distribution of brain conventional MRI findings in 24 NPSLE and 45 SLE patients according to the presence of brain-reactive autoantibodies detected using IHC.

Brain MRI findings	Positive brain-IHC (n=42)	Negative brain-IHC (n=27)	p
**Abnormal MRI**	24 (57.1%)	15 (37.0%)	0.897
**Acute lesions**	2 (8.3%)	4 (14.8%)	0.151
** Inflammatory type lesions**	2 (8.3%)	2 (7.4%)	0.648
** Myelopathy**	2 (8.3%)	1 (3.7%)	0.834
** Large infarct**	0	0	
**Chronic lesions**	24 (57.1%)	13 (48.1%)	0.467
** WMHIs**	23 (54.8%)	12 (44.4%)	0.306
** GMHIs**	5 (11.9%)	0	0.064
** Diffuse brain atrophy**	1 (2.4%)	4 (14.8%)	0.053
** Areas of resolved CVA**	4 (9.5%)	1 (3.7%)	0.366

MRI, magnetic resonance imaging; WMHIs, white matter hyperintensities; GMHIs, grey matter hyperintensities; CVA, cerebral-vascular accident (including large infarcts, lacunar lesions, haemorrhages).

### Sensitivity analysis in subpopulation of NPSLE and SLE patients

The diagnostic performance of brain-reactive autoantibodies, excluding anti-P and anti-NR2, in distinguishing between NPSLE and SLE patients was unsatisfactorily with 75.0% (95%CI 57.8% to 87.9%) sensitivity and 44.9% (95%CI 32.9% to 57.4%) specificity, resulting in 41.5% (95%CI 29.4% to 54.4%) positive predictive value (PPV) and 77.5% (95%CI 61.5% to 89.2%) negative predictive value (NPV). More stringent definition of NPSLE and SLE, by respectively excluding the 5 patients with headache and the 6 ANA negative patients at the time of sampling, gave similar results in term of sensitivity (74.2%; 95%CI 55.4% to 88.1%), specificity (46.0%; 95%CI 33.4% to 59.1%), PPV (40.4%; 95%CI 27.6% to 54.2%) and NPV (78.3%; 95%CI 61.8% to 90.2%). A positivity threshold ≤1:480 for brain-reactive antibodies led to higher specificity (75.4%; 95%CI 63.5% to 85.0%) but lower sensitivity (58.3%; 95% CI 40.8% to 74.5%).

## Discussion

The present study revealed that NPSLE and SLE patient sera might carry autoantibodies targeting the entire brain, especially the hippocampus, cerebellum, and cortex. Furthermore, brain-reactive autoantibodies against known and unknown antigens were found more frequently and with higher intensity and titers in NPSLE, suggesting their possible role in the pathogenesis of NPSLE. The exclusive finding of GMHIs on conventional brain MRIs from NPSLE patients positive for brain-reactive autoantibodies may support this hypothesis. Previous studies reported an inflammatory immune response mediated by autoantibodies to brain antigens as responsible for GMHIs (23). In contrast, vascular changes mediated by accelerated atherosclerosis are claimed as responsible for brain atrophy (24, 25), which may suggest an underlying alternative pathogenetic mechanism in those NPSLE patients that were negative for brain-reactive autoantibodies. On the other hand, the high percentage of SLE patients carrying brain-reactive autoantibodies without neurologic symptoms might suggest they lack a pathogenetic effect. However, the complement cascade activation demonstrated by the significantly lower serum levels of C3 and C4 observed in our cohort of NPSLE patients may provide indirect evidence of a pathogenetic effect of brain-reactive antibodies in this group. Complement cascade activation is primarily responsible for BBB and B-CSF disruption (26), and it may account for higher circulating brain-reactive antibodies access to the CNS environment in NPSLE than in SLE patients. Brain-reactive autoantibodies need to access the CNS environment *in vivo* through a blood-brain barrier (BBB) or blood-cerebrospinal fluid (B-CSF) barrier leakage to bind neuronal cells, causing apoptotic cell death, microglial activation, synaptic pruning, and reduced synaptic density (27, 28). Unfortunately, for ethical reasons, we could not check for brain-reactive autoantibodies positivity in CSF, which represents a major limitation of our study.

In the preliminary stage of this study, we investigated whether the rat-IHC positive sera included known autoantibodies (e.g., ANA) and were directed against defined antigens (e.g., anti-Sm, anti-P, anti-NR2). Although ANA can target any epitope within the nucleus, regardless of the cellular type used, we did not reveal any immunostaining reaction using rat brain sections in 44.9% and 25% of ANA HEp-2 positive sera collected from SLE and NPSLE, respectively. Furthermore, double staining experiments using the rat-IHC positive sera and commercial antibodies that mark neurons, astrocytes, and oligodendrocytes showed that brain-reactive autoantibodies labeled only neurons and not glial cells. If we could have detected by IHC the same ANA revealed through HEp-2, such autoantibodies would have labeled both neuron and glial cells. That could be explained by the differences in detecting ANA according to the substrates used because cell-based assays could reveal different epitopes than tissue-based assays. Among the known autoantibodies tested, only the anti-β2GPI were detected in the rat-positive IHC sera and were virtually absent in the negative ones suggesting they may be part of the heterogeneous autoantibody population responsible for the labeling we observed in rat brains. Indeed, the aPL and especially the anti-β2GPI could target neurons of the human brain, and patients with these autoantibodies were found to be more prone to exhibit seizures, cognitive impairment, psychosis, and depression compared to seronegative ones (15, 29, 30).

Finally, we tried to find a potential neuronal target for brain-reactive autoantibodies. The fact that the autoantibodies labeled NeuN-containing cells within the entire brain lead us to believe that NeuN might be one of the possible main autoantigens recognized by the autoantibodies. NeuN is a neuronal nuclear protein with a mass of around 46/48 kDa and is coded by the RNA Binding Fox-1 Homolog 3 (*RBFOX3*) gene (31). It plays an essential role in regulating the transcription and RNA processing of many genes involved in the maturation of neurons (32). NeuN is widely used as a post-mitotic neuronal marker, and only a few neurons do not express this protein, such as the Purkinje cells (33, 34). Moreover, *RBFOX3* mutations are linked to epilepsy (35) and cognitive impairments (36), while *RBFOX3* knockout mice have a higher risk of developing seizures and anxiety-related behavior disorders (37). Thus, our hypothesis that autoantibodies recognized NeuN was also compatible with its involvement in neuropsychiatric symptoms. Furthermore, NeuN localizes in most neurons’ nuclei or perinuclear cytoplasm. Due to these reasons, it is considered the perfect marker for staining neurons (38). However, we proved that NeuN was not the autoantibody target.

The present study has several limitations. First, we did not test the positivity of brain-reactive antibodies in the CSF from NPSLE and SLE patients. The ethical reason behind this limitation lies in the observational design of the present study, which prevented us from performing a lumbar puncture to collect CSF in SLE patients without neuropsychiatric manifestations and in mild to moderate NPSLE patients for whom such an invasive procedure was considered unnecessary. Therefore, confirming the presence of brain-reactive autoantibodies in the CSF represents an important future perspective. Second, we did not identify a new autoantibodies specificity in the brain, and we did not perform functional investigations, which are a primary focus in NPSLE research. Since the target antigens were intraneuronal, proving brain-reactive autoantibodies can perturb neuronal function would be paramount to confirm their pathogenetic effect in NPSLE patients. On the other hand, the general cytosolic pattern we observed in IF and TEM does not exclude cross-reactivity with neuronal membrane proteins, as lately demonstrated for the anti-P reacting with the neuronal surface antigen (10) and for anti-dsDNA reacting with NR2 (11) to exert their pathogenetic effects. Future experiments should investigate the functional effect of brain-reactive antibodies on brain function *in vitro* and *in vivo* through advanced imaging techniques. Furthermore, testing the effect of incorporating brain-reactive antibodies in the performance of algorithms for attributing neuropsychiatric events in SLE would be a further important point in the research agenda (39).

In conclusion, the data obtained from this study demonstrate that (a) SLE and NPSLE patients could possess autoantibodies potentially reacting to neuronal cells, with higher frequency and titers in NPSLE according to the broad spectrum of the neurological symptoms; (b) the whole brain but in particular the cortex, the hippocampus, and the cerebellum could be the target of brain-reactive autoantibodies mainly directed against unknown antigens; (c) anti-β2GPI could be part of the autoimmune reaction occurring at the brain level. However, further studies are needed to disentangle the specificity of brain-reactive autoantibodies in SLE patients.

## Data availability statement

The raw data supporting the conclusions of this article will be made available by the authors, without undue reservation.

## Ethics statement

All national and institutional guidelines for the care and use of animals were followed. Experimental protocols were performed in agreement with the Italian legislation, while the care and use of animals were approved by the American Physiological Society and EEC council directive of 24 November 1986 (86/609). Furthermore, the Independent Ethical Committee AOU of Cagliari (protocol number PG/2019/4522) approved the animal and human experimental design and procedures and all the subjects provided written informed consent.

## Author contributions

Study conception: CC, GF, AM, MP. Study design: CC, GF, AM, MP. Acquisition, analysis, or interpretation of the data: EM, CC, GC, MA, BN, MC, FL, MI, EC, AF, LL, LS, AC. Drafting the manuscript: CC, EM, MA, MP. Substantial manuscript revision: EM, GC, MA, MC, FL, MI, EC, AF, LL, LS, GF, CC, AM, AC. All authors read and approved the final manuscript.

## Supplementary material

Supplementary Figure 1 In the cerebellum’s coronal sections, the autoantibodies’ staining patterns (**A, E, I**: red labeling) were compared with the anti: -GAD, vGlut1, and VAChT (**B, F, J**, respectively, green labeling). Colocalization was not observed when mixing the anti-GAD, vGlut1, and VAChT antibodies with autoantibodies (merge **C, G, K**, red labeling + green labeling), as shown by Pearson’s coefficient (always <0.5). In addition, autoantibody labeling was identified with Cy3 red labeling, while the antibodies were identified with Alexa488 green labeling. GAD: glutamic acid decarboxylase; vGlut1 vesicular glutamate transporter 1, VAChT: Vesicular Acetylcholine Transporter. Scale bar: 0,1 mm.
